# Prevalence and social inequalities in the use of cancer screening tests in Campinas, Brazil (ISACamp 2014/15)

**DOI:** 10.1590/1980-549720250043

**Published:** 2025-08-04

**Authors:** Bianca Gastaldon Lima, Marilisa Berti de Azevedo Barros

**Affiliations:** I Universidade Estadual de Campinas, Faculty of Medical Sciences, Department of Collective Health - Campinas (SP), Brazil.

**Keywords:** Mass screening, Cancer, Men’s health, Women’s health

## Abstract

**Objective:**

To analyze the prevalence of early detection tests for cervical, breast, prostate, and colorectal cancers in the population of Campinas, São Paulo and the presence of social inequalities in access.

**Methods:**

Population-based cross-sectional study using data from ISACamp 2014/15. Dependent variables were the performance of Pap smear, mammogram, prostate-specific antigen (PSA), fecal occult blood test (FOBT) and colonoscopy/sigmoidoscopy within the age ranges recommended by national guidelines. Independent variables included sex, age, schooling, income, race/skin color, and private health insurance. Prevalence and prevalence ratio adjusted for sex and age were estimated using Poisson regression. Analyses were performed using Stata 14, considering sampling weights.

**Results:**

The prevalence of mammography (77.7%) and Pap smear (87.8%) met the targets set by the Brazilian Ministry of Health, while FOBT (22.3%) and colonoscopy/sigmoidoscopy (21.5%) showed low coverage. PSA testing in the previous three years was reported by 55.2% of eligible men. Higher prevalence of test performance was observed among individuals with higher schooling and income levels and those with private health insurance. Inequalities varied by type of test. For instance, individuals with private health insurance had 11 and 162% higher prevalence of Pap test and colonoscopy/sigmoidoscopy, respectively, compared to those without insurance. Racial inequality was observed only for mammography.

**Conclusion:**

The results indicate high coverage and lower inequalities for Pap and mammography, and low coverage with significant disparities for FOBT and colonoscopy/sigmoidoscopy. Findings highlight the need to monitor coverage and to implement public policies aimed at reducing inequities in access to cancer screening.

## INTRODUCTION

In recent decades, cancer has emerged as the second leading cause of death globally, behind only cardiovascular diseases^1^ ^,^ ^2^. In some European countries and Canada, it is already the leading cause of death^3^.

Due to the high lethality rate, early detection is essential and can be achieved through screening, which applies tests to asymptomatic individuals to identify cancer at an early stage, allowing for less invasive treatments and longer survival^4^. However, screening involves risks, such as false positives, false negatives, overtreatment and overdiagnosis^5^.

The World Health Organization (WHO) and the International Agency for Research on Cancer (IARC) recommend screening for cervical, breast and colorectal cancer, due to strong evidence of benefits^6^ ^,^ ^7^. However, for prostate cancer, these institutions do not recommend routine screening in asymptomatic men, given the lack of evidence on the risks and benefits of the prostate-specific antigen (PSA) test^5^ ^,^ ^6^ ^,^ ^7^ ^,^ ^8^.

Studies show that the prevalence of screening tests varies between countries and there are social inequalities in access^9^ ^,^ ^10^ ^,^ ^11^ ^,^ ^12^ ^,^ ^13^ ^,^ ^14^ ^,^ ^15^ ^,^ ^16^ ^,^ ^17^ ^,^ ^18^ ^,^ ^19^ ^,^ ^20^ ^,^ ^21^. In Brazil, the number of cervical and breast examinations has increased, but with inequality between socioeconomic groups^20^ ^,^ ^21^ ^,^ ^22^ ^,^ ^23^ ^,^ ^24^ ^,^ ^25^ ^,^ ^26^.

Given the importance of early detection and disparities in access, this study analyzed the prevalence of Pap smears, mammography, PSA, fecal occult blood test (FOBT) and sigmoidoscopy/colonoscopy in the population of Campinas (SP) and the social inequalities present in access to these examinations.

## METHODS

We conducted a cross-sectional population-based study developed with data from the 2014/2015 Campinas Municipal Health Survey (ISACamp), which aims to monitor health indicators and use of services in three age groups: adolescents, adults and the elderly. The survey was conducted in a sample of the non-institutionalized population living in private households in the urban area of ​​the municipality of Campinas.

The sampling was probabilistic, stratified, by clusters and carried out in two stages. In the first stage, 70 census tracts were randomly selected, and in the second, households in these 70 tracts were selected to compose independent samples of 1,000 adolescents (10-19 years old), 1,400 adults (20-59 years old) and 1,000 older people (60 years old or older).

These sample sizes allowed us to estimate a proportion of 0.5, with a significance level of 95%, a sampling error of 4 or 5 percentage points, considering a design effect of 2. The number of households selected for each age domain were 3,119, 1,029 and 3,161, respectively. The ISACamp 2014/15 data collection was carried out using tablet devices by trained and supervised interviewers. The questionnaire used consists of 12 thematic blocks, with most of the questions closed, and is applied directly to the selected individual.

In this study, only data from adults and older people were used, in the recommended age groups for the early detection examinations analyzed.

The dependent variables used in this study were:

- Pap smear screening for cervical cancer. This was analyzed in the three years prior to the interview for women aged 25 to 64, the age range recommended by the Ministry of Health;
- Mammography screening for breast cancer. This was analyzed in the two years prior to the interview for women aged 40 to 69, the age range recommended by the Municipal Secretariat of Campinas, and for women aged 50 to 69, the age range recommended by the Ministry of Health;
- PSA screening for prostate cancer. Two variables were analyzed: screening for colorectal cancer in the three years prior to the interview and at least once in life for men aged 50 or over, as recommended by the Brazilian Society of Urology;
- FOBT for colorectal cancer screening in the two years prior to the interview, in men and women aged 50 or over, as recommended by the Brazilian Society of Coloproctology and the Ministry of Health;
- Performing a sigmoidoscopy or colonoscopy at least once in a lifetime to screen for colorectal cancer, in men and women aged 50 or over, as recommended by the Brazilian Society of Coloproctology and the Ministry of Health.

The independent variables analyzed were: sex (male and female), age group (in years), education (in years of completed study: 0 to four, five to 11, and 12 or more years), affiliation with private health insurance (with or without a plan), self-reported race/skin color (white, black and brown - Asian and indigenous people were not included in the analysis because of the small number in the sample), and monthly *per capita* family income (in minimum wages: <1, 1 to <2, and 2 or more).

The data were analyzed in Stata 14 (StataCorp, College Station, TX, United States) using the survey module, suitable for complex samples. The prevalence of preventive practices was estimated with a 95% confidence interval (95%CI). Associations between variables were verified by Pearson’s χ2 test (p≤0.05). Prevalence ratios between sociodemographic segments were estimated and compared using Poisson multiple regression with robust variance, adjusted for sex and age.

ISACamp 2014/15 was approved by the Ethics Committee of the School of Medical Sciences of the State University of Campinas (Opinion No. 409.714/2013), as was the present project (Opinion No. 6.983.946/2024).

### Data availability statement

The data used in this study are available upon request to the corresponding author, considering the privacy and confidentiality criteria of the participants, in accordance with the applicable ethical standards.

## RESULTS

Table 1 givess the prevalence of early detection tests and the number of people in the sample (n) in the recommended age range. The results show that in Campinas, the prevalence of Pap smears (in the three years prior to the interview) was 87.8% among women aged 25 to 64; and mammography (in the two previous years) was 80.5% among women aged 40 to 69 and 77.7% among those 50 to 69.

**Table 1.** Prevalence (95%CI) of tests for early detection of cancer. Health Survey of the Municipality of Campinas 2014/15.

Tests performed	Age group (years)	n	% prevalence (95%CI)
Pap smear (in last three years)	25 to 64	639	87.8 (84.2-90.7)
Mammography (in last two years)	40 to 69	548	80.5 (75.1-84.9)
Mammography (in last two years)	50 to 69	315	77.7 (71.5-82.9)
PSA (in last three years)	50 or older	499	55.2 (49.7-60.5)
PSA (once in a lifetime)	50 or older	499	80.1 (75.0-84.4)
FOFT (in last two years)	50 or older		
Both sexes		1,228	22.3 (17.8-27.5)
Men		499	22.2 (17.1-28.3)
Women		729	22.4 (17.5-28.2)
Sigmoidoscopy/colonoscopy (once in a lifetime)	50 or older		
Both sexes		*1,217	21.5 (17.9-25.5)
Men		495	17.6 (13.0-23.3)
Women		722	24.6 (20.5-29.2

95%CI: 95% confidence interval; PSA: prostate-specific antigen; FOBT: fecal occult blood test; n: total number of individuals in each age group, regardless of tests performed; *11 people did not respond.

In Campinas, 55.2% of men aged 50 or over had a PSA test in the three years prior to the interview, and 80.1% had it at least once in their lifetime. Among the population aged 50 or over, 22.3% underwent the FOBT in the previous two years, with no difference in prevalence between sexes, and 21.5% underwent colonoscopy/sigmoidoscopy at least once in their life, with a significantly higher prevalence in females (24.6%) compared to males (17.6%) (Table 1), with an age-adjusted prevalence ratio (PR) of 1.38 (1.02-1.85).

In the analyses of differences in prevalence according to sociodemographic characteristics, a higher prevalence of screening tests was observed among people with health insurance. The inequality was small for Pap smear (PR=1.11) and mammography (PR=1.20-1.29), intermediate for PSA (PR=1.47) and FOBT (PR=1.67), and very high for colonoscopy/sigmoidoscopy (PR=2.62) (Table 2).

**Table 2.** Prevalence and prevalence ratio* (95%CI) of tests performed for early detection according to having private health insurance. Health Survey of the Municipality of Campinas 2014/15.

Tests performed	No insurance	Insurance	PR (95%CI)**
Pap smear (in last three years)	83.0 (77.1-87.5)	92.9 (88.5-95.6)	1.11 (1.04-1.20)
Mammography^a^ (in last two years)	73.3 (66.6-79.1)	87.9 (80.9-92.6)	1.20 (1.08-1.32)
Mammography^b^ (in last two years)	68.0 (60.0-75.0)	88.0 (79.8-93.1)	1.29 (1.13-1.47)
PSA (in last three years)	46.0 (39.0-53.0)	68.0 (58.4-76.2)	1.47 (1.20-1.78)
PSA (once in a lifetime)	73.7 (66.9-79.6)	89.2 (82.5-93.6)	1.20 (1.09-1.32)
FOBT (last two years)			
Both sexes	16.9 (12.3-22.9)	28.5 (21.7-36.4)	1.67 (1.13-2.46)
Men	18.4 (12.7-25.8)	27.4 (20.1-36.2)	1.46 (0.96-2.22)
Women	15.5 (10.8-21.7)	29.2 (21.2-38.7)	1.87 (1.16-3.01)
Sigmoidoscopy or colonoscopy (once in a lifetime)			
Both sexes	12.2 (9.5-15.6)	32.3 (25.8-39.5)	2.62 (1.88-3.65)
Men	10.8 (7.5-15.3)	27.1 (18.6-37.7)	2.45 (1.54-3.90)
Women	13.4 (10.0-17.8)	35.6 (28.2-43.8)	2.64 (1.78-3.92)

*Adjusted for sex and age; ^a^women aged 40 to 69 years; ^b^women aged 50 to 69 years; 95%CI: 95% confidence interval; PSA: prostate-specific antigen; FOBT: fecal occult blood test; PR: prevalence ratio; **population with health insurance/population without health insurance and white population/black and brown population.

Table 3 shows that the prevalence rates are significantly higher among individuals with 12 or more years of schooling, with no statistically significant difference between those with intermediate and lower levels of schooling, except for PSA (one test in life), which was 16% higher among those with five to 11 years of schooling compared to those with less than four years. The greatest inequalities occurred in FOBT (PR=2.20) and colonoscopy/sigmoidoscopy (PR=2.86) tests.

**Table 3:** Prevalence and prevalence ratio* (95%CI) of early cancer detection tests according to years of schooling. Health Survey of the Municipality of .Campinas 2014/15.

Tests performed	0 to four years of schooling	Five to 11 years of schooling	12 or more years of schooling	PR (95%CI) (5 to 11/≤4)	PR (95%CI) (12 or +/≤4)
Pap smear (in last three years)	81.7 (73.1-88.0)	86.9 (82.3-90.5)	92.8 (86.5-96.3)	1.10 (0.99-1.22)	1.18 (1.07-1.31)
Mammography^a^ (in last two years)	71.4 (62.7-78.8)	80.6 (73.4-86.2)	92.4 (81.3-97.1)	1.12 (0.97-1.29)	1.28 (1.08-1.50)
Mammography^b^ (in last two years)	68.3 (59.8-75.8)	79.1 (70.4-85.7)	94.1 (82.3-98.2)	1.15 (0.97-1.34)	1.36 (1.15-1.60)
PSA (in last three years)	54.2 (47.0-61.2)	51.8 (43.8-59.6)	64.5 (49.2-77.4)	1.12 (0.90-1.38)	1.38 (1.06-1.79)
PSA (once in a lifetime)	75.3 (67.2-82.0)	79.0 (71.2-85.1)	92.5 (79.7-97.5)	1.16 (1.01-1.34)	1.36 (1.16-1.58)
FOBT (in last two years)					
Both sexes	20.4 (15.5-26.3)	18.0 (13.0-24.3)	37.6 (24.7-52.5)	1.05 (0.75-1.45)	2.20 (1.42-3.44)
Men	21.4 (15.9-29.1)	17.5 (11.5-25.6)	33.7 (20.7-49.8)	1.12 (0.69-1.83)	2.15 (1.25-3.67)
Women	19.6 (14.5-26.0)	18.4 (12.7-25.9)	41.5 (22.3-63.6)	1.01 (0.64-1.59)	2.31 (1.30-4.10)
Sigmoidoscopy or colonoscopy (once in a lifetime)					
Both sexes	19.1 (14.8-24.1)	14.1 (11.1-17.7)	46.1 (32.2-60.7)	0.87 (0.63-1.20)	2.86 (1.88-4.36)
Men	15.5 (11.5-20.6)	9.6 (5.6-15.8)	39.7 (23.8-58.0)	0.9 (0.51-1.55)	3.61 (2.15-6.06)
Women	21.4 (15.8-28.2)	18.0 (13.2-23.9)	52.8 (34.1-70.7)	0.89 (0.57-1.38)	2.63 (1.61-4.31)

*Adjusted for sex and age; ^a^women aged 40 to 69 years; ^b^women aged 50 to 69 years; 95%CI: 95% confidence interval; PSA: prostate-specific antigen; FOBT: fecal occult blood test; PR: prevalence ratio.

Table 4 displays the PRs of the tests according to race/skin color. It is observed that there were no significant differences for four of the examinations analyzed, and only the prevalence of mammography was significantly higher among white women aged 40 to 69 years (PR=1.17) compared to the black population.

**Table 4.** Prevalence and prevalence ratio* (95%CI) of early detection tests according to race/skin color. Health Survey of the Municipality of Campinas 2014/15.

Tests performed	Blacks and browns	Whites	PR (95%CI)**
Pap smear (in last three years)	85.3 (80.1-89.4)	89.2 (84.9-92.5)	1.04 (0.98-1.11)
Mammography^a^ (in last two years)	72.4 (62.0-80.8)	84.1 (78.6-88.4)	1.17 (1.02-1.33)
Mammography^b^ (in last two years)	71.1 (60.5-79.9)	81.0 (74.0-86.5)	1.14 (0.98-1.32)
PSA (in last three years)	53.2 (43.4-62.7)	56.4 (49.8-62.9)	1.07 (0.86-1.35)
PSA (once in a lifetime)	77.6 (65.7-86.2)	81.7 (75.3-86.8)	1.05 (0.90-1.24)
FOBT (in last two year			
Bothe sexes	20.6 (15.4-26.8)	23.2 (17.9-29.4)	1.12 (0.82-1.53)
Men	19.8 (13.4-28.4)	23.1 (17.3-30.1)	1.19 (0.81-1.75)
Women	21.1 (14.5-29.6)	23.2 (17.0-30.7)	1.09 (0.68-1.75)
Sigmoidoscopy or colonoscopy (once in a lifetime)			
Both sexes	20.1 (15.1-26.3)	22.0 (17.6-27.1)	1.08 (0.76-1.55)
Men	18.5 (10.9-29.8)	17.2 (11.8-24.4)	0.95 (0.49-1.82)
Women	21.3 (15.5-28.6)	25.8 (20.5-31.9)	1.20 (0.80-1.81)

*Adjusted for sex and age; ^a^women aged 40 to 69 years; ^b^women aged 50 to 69 years; 95%CI: 95% confidence interval; PSA: prostate-specific antigen; FOBT: fecal occult blood test; PR: prevalence ratio; **population with health insurance/population without health insurance and white population/black and brown population.

Table 5 shows a higher prevalence of all early detection tests among individuals with a *per capita* family income higher than two minimum wages, with colonoscopy/sigmoidoscopy being notable at 2.5 times more prevalent. Individuals with an income of one to two minimum wages had a higher prevalence of mammography (women aged 40 to 69 years) and colonoscopy/sigmoidoscopy compared to those with an income lower than one minimum wage.

**Table 5.** Prevalence and prevalence ratio.* (95%CI) of early detection tests according to ***per capita*** family income in minimum wages. Health Survey of the Municipality of Campinas 2014/15.

Tests performed	<1 MW	1 to <2 MW	2 or more	PR (95%CI) (1 to <MW/<1)	PR (95%CI) (2 or +/<1)
Pap smear (in last three years)	82.7 (75.7-88.0)	89.3 (83.4-93.2)	92.2 (87.9-95.1)	1.08 (0.98-1.18)	1.11 (1.03-1.2)
Mammography^a^ (in last two years)	73.4 (66.2-79.6)	80.5 (73.0-86.3)	86.5 (77.6-92.2)	1.10 (1.0-1.21)	1.19 (1.06-1.34)
Mammography^b^ (in last two years)	66.2 (56.7-74.6)	75.5 (66.2-82.9)	88.3 (78.7-93.9)	1.14 (0.97-1.34)	1.34 (1.16-1.55)
PSA (in last three years)	46.7 (36.8-56.9)	54.0 (44.1-63.6)	63.2 (53.5-71.9)	1.15 (0.86-1.52)	1.36 (1.04-1.77)
PSA (once in a lifetime)	72.4 (60.8-81.7)	78.8 (68.8-86.2)	87.2 (79.2-92.4)	1.08 (0.89-1.31)	1.20 (1.01-1.42)
FOBT (in last two years)					
Both sexes	16.3 (11.8-22.2)	21.0 (15.3-28.0)	28.0 (20.1-37.5)	1.29 (0.9-1.8)	1.73 (1.11-2.7)
Men	14.4 (8.2-23.8)	23.5 (15.7-33.6)	25.7 (18.7-34.1)	1.60 (0.91-2.82)	1.81 (1.03-3.17)
Women	17.7 (11.9-25.4)	18.7 (13.0-26.2)	29.8 (19.8-42.3)	1.06 (0.64-1.73)	1.69 (0.92-3.07)
Sigmoidoscopy or colonoscopy (once in a lifetime)			
Both sexes	11.6 (8.0-16.4)	21.4 (15.8-28.4)	28.8 (21.7-37.2)	1.85 (1.14-3.0)	2.50 (1.58-3.98)
Men	6.8 (3.31-13.3)	16.8 (10.5-25.7)	25.2 (17.6-34.7)	2.41 (1.08-5.36)	3.78 (1.79-8.00)
Women	14.9 (9.8-21.9)	25.4 (18.8-33.3)	31.7 (22.1-43.1)	1.70 (1.00-2.90)	2.13 (1.19-3.78)

*Adjusted for sex and age; ^a^women aged 40 to 69 years; ^b^women aged 50 to 69 years; 95%CI: 95% confidence interval; PSA: prostate-specific antigen; FOBT: fecal occult blood test; PR: prevalence ratio; MW: minimum wage.

## DISCUSSION

The results of this study indicated that the prevalence of Pap smears and mammograms in Campinas reached the targets recommended by the Ministry of Health. The prevalence of FOBT and colonoscopy/sigmoidoscopy was lower than that of other prevalences. The analyses revealed social inequalities in access to screening, with a higher prevalence among individuals with higher levels of education and income and with health insurance. A race-color disparity was observed in mammography screening, with a higher prevalence among white women, and a sex disparity in colonoscopy/sigmoidoscopy screening, with a higher prevalence among women compared to men (PR=1.38).

Cervical cancer screening coverage in Campinas was 87.8%, reaching the 85% target recommended by the Ministry of Health^27^. In the city of São Paulo (SP), in the same period, coverage was similar, reaching 89.6%^23^. Data from the National Health Survey (PNS) showed an increase in the rate of Pap smears performed by Brazilian women from 78.7% in 2013 to 81.3% in 2019^20^ but with a percentage still slightly below that recommended by the Ministry of Health.

Data from studies conducted in some developed countries show similar or lower prevalence rates for this test than in the Brazilian population. In the United Kingdom, the prevalence of Pap smears among women aged 25 to 64 was 71% in 2018^10^, and in the United States, among women aged 21 to 65, it was 80% in 2018^9^.

Previous studies conducted in the city of Campinas show that Pap smears continue to be performed at a high level and with a slight upward trend: 83.3% in 2001/2002, 86.2% in 2008/09 (both in women aged 20 to 60), and 87.8% in the present study (in women aged 25 to 64)^21^ ^,^ ^22^.

In the present study, the prevalence of the test was 11% higher in the segment with the highest income, 18% in the segment with the highest level of education and 11% in women with health insurance, compared to the reference categories. The inequalities observed in the Brazilian population, with data from the 2019 PNS, were: 24% higher in the segment with the most years of education, 29% higher in the highest income level and 20% higher in women with health insurance^20^, slightly higher therefore than those observed in Campinas.

A study carried out in 22 European countries found that the prevalence of the Pap smear was on average 28% higher in the segments of women with the highest level of education, ranging from 7.7% in Luxembourg to 44% in Croatia^12^. The inequalities according to level of education observed in Campinas and in Brazil are therefore below the average detected for these European countries.

In Campinas, a study developed with data from the 2008/2009 survey had not detected social inequalities in access to Pap smears among women aged 20 to 60^22^. The resurgence of inequality shows the need to monitor not only the coverage of exams, but also the presence and size of social inequalities in their performance.

For mammograms, the Ministry of Health recommends coverage of 70% for women aged 50 to 69 years^27^. In the city of Campinas, the present study detected a prevalence of 77.7% in this age group, exceeding the recommended target, which was also observed in the city of São Paulo, which found a prevalence of 73.8% in 2015^23^. Data from the 2013 PNS showed that 79.4% of women aged 40 to 70 years had had a mammogram in the two years prior to the interview; however, in the age group recommended by the Ministry of Health, the prevalence was 58%^25^. A study conducted in 2010 in 22 European countries revealed that the prevalence of mammography in countries with organized screening ranged from 69.8% in the United Kingdom to 87.9% in Finland and, among the four countries without organized screening, from 38.1% in Latvia to 50.5% in the Czech Republic^12^. In the United States, the prevalence of mammograms was 78% in 2020, ranging from 66 to 87% among American states^11^.

Comparing the prevalence of mammography in Campinas with data from other countries, it was found that, even without organized screening for breast cancer, the prevalence rates are similar and even higher than in some countries that have organized screening.

In the present study, the prevalence rates for women aged 50 to 69 years were: 36% higher in the segment with the highest level of schooling, 34% in the segment with the highest income and 29% among women with health insurance compared to the reference categories. Data from the 2013 PNS showed a prevalence twice as high in the segment with the highest level of education and 11% higher among women with health insurance^25^ ^,^ ^28^. Inequalities in mammograms, measured by level of education, vary significantly among some European countries: 46.4% in Greece, 47.9% in Croatia and 82.8% in the Czech Republic^12^, which have inequalities greater than those observed in Campinas.

Routine screening for prostate cancer using PSA testing is not recommended for asymptomatic men by the WHO and IARC due to the lack of clear evidence on the benefits and risks^6^ ^,^ ^7^. The Ministry of Health also advises against screening, while the Brazilian Society of Urology recommends that men aged 50 or over discuss with their urologists the possibility of undergoing early detection tests for prostate cancer^5^ ^,^ ^29^.

The present study detected a prevalence of 55.2% of men aged 50 or over having been tested for PSA in the three years prior to the survey, and 80.1% of men aged 50 or over having had the test at least once in their lifetime. A study conducted in the city of São Paulo during the same period found that 63.2% of men aged 40 or over had had the PSA test at least once in their lifetime^23^. The prevalence of having had the PSA test at least once in their lifetime was 70% among men aged 55 to 69 in a study conducted in the Netherlands^16^, and 44.2% among men aged 40 or over in a study conducted in Portugal^17^. Although different age groups were analyzed, having had the PSA test once in a lifetime in Campinas was higher than in these countries and than in the city of São Paulo. In the present study, the number of men with higher incomes undergoing PSA assay in the three years prior to the interview was 36% higher, while in men with higher levels of education, this was 38% higher and 47% higher with health insurance. A study conducted in the city of São Paulo in 2015 found that the number of men aged 40 or over undergoing PSA testing once in a lifetime was 48% higher in the segment with higher levels of education^23^, an inequality that does not differ significantly from that observed in Campinas.

The results of this study indicate that, even without government guidelines for population screening for prostate cancer, a significant portion of men in Campinas underwent PSA testing, exceeding the prevalence observed in European countries and in the city of São Paulo. However, inequalities in access to the test persist, with higher rates among men with higher socioeconomic status.

The Ministry of Health recommends screening tests for colorectal cancer, but does not have an organized screening program or establish specific coverage targets for each test^5^ ^,^ ^27^. European Union guidelines establish coverage of over 65% for FOBT every two years as a guarantee of quality in colorectal cancer screening^15^.

The results of this study showed prevalence rates of 22.3% for FOBT performed in the two years prior to the interview and 21.5% for colonoscopy/sigmoidoscopy (one examination in a lifetime) among individuals aged 50 years or older. Data from 2007 from European countries revealed that rates of taking FOBT, using fecal immunochemical testing every two years, ranged from 42% in France to 70.8% in Finland^14^. A 2019 study developed with data from the National Health Interview Survey revealed that, among seniors aged 65 to 75 years in the United States, 19.2% had undergone colonoscopy and 6% had FOBT in the year prior to the survey^13^.

A systematic review that analyzed 96 articles from 14 countries found wide variation in the population’s participation in colorectal cancer screening tests, with prevalence rates for sigmoidoscopy ranging from 7 to 76.1%, for FOBT ranging from 2.3 to 68.7%, and for colonoscopy after a positive FOBT result ranging from 72.9 to 92.6%^19^.

Comparing prevalence rates across studies is difficult due to differences in age groups and the period in which the tests are performed, as well as the lack of national studies analyzing the prevalence of early colorectal cancer detection tests. However, comparison with data from other countries indicates low coverage of these tests in the city of Campinas.

Another relevant finding was the presence of high inequalities in access to these tests. FOBT was 73% more frequent among individuals with higher incomes, and colonoscopy/sigmoidoscopy was 150% more common in this group. In terms of education, access to FOBT was 120% higher among those with more education, while colonoscopy/sigmoidoscopy showed a difference of 186%. In individuals with health insurance, the prevalence of FOBT was 67% higher, and that of colonoscopy/sigmoidoscopy was 162% higher.

The systematic review that focused on social inequalities in participation in colorectal cancer screening programs found that, in general, segments of the population with higher levels of education and income have greater participation in FOBT and colonoscopy^19^. A Danish study from 2010 revealed an odds ratio of 1.38 in performing FOBT among those with higher education compared to those with less education^30^. Despite the difference in the period analyzed and in the way the association was measured, the inequality observed in Denmark was much lower than that observed in Campinas in the present study.

Among the results of this study, it was found that colonoscopy/sigmoidoscopy was 38% higher in women compared to men, with no difference between the sexes in getting FOBT. Unlike our study, articles from other countries have shown a higher prevalence of FOBT in women compared to men^19^ ^,^ ^30^. In the aforementioned meta-analysis, most studies detected greater participation of women, compared to men, in colorectal cancer screening, which is attributed to their greater use of health services and greater familiarity with screening programs^19^. While self-care is more present in women, to prevent personal and family suffering, men have a poor perception of vulnerability^19^.

The results of this study revealed low coverage of early detection exams for colorectal cancer in Campinas and significant socioeconomic inequalities in the performance of these exams, which highlights the need for adequate structuring of health services to expand the provision of exams and reduce inequalities in access.

The present study is relevant because it analyzed, jointly, several early detection examinations for cancer, allowing comparisons regarding the population coverage achieved and the magnitude of the associated social inequalities. Among the limitations of the study, it is worth pointing out the possibility of memory and information bias, since the data were obtained through interviews, and the interviewees may have been confused about the time at which they had the test and may have responded that they had it because they considered this to be the best answer, as indicated in other studies^24^ ^,^ ^25^ ^,^ ^26^.

To mitigate these biases, this study was conducted with methodological rigor, including the application of standardized questionnaires and specific training of interviewers to ensure the clarity of questions and reduce ambiguous interpretations. Since this was a cross-sectional study, it is also necessary to consider that the questions about the performance of examinations refer to the two or three years prior to the interview or to some time in life, the question about income refers to the previous month and the question about education and having health insurance at the time of the interview.

This study highlights the importance of monitoring the performance of examinations for early detection of cancer to determine the degree to which the established goals are being achieved. By highlighting and measuring disparities in access to screening between socioeconomic segments, it makes it possible to identify the examinations with a lower or higher degree of inequality of access and the segments of the population at greater risk of late diagnosis and guide measures that promote advances in health equity. The study also generates information for future comparisons with post-pandemic data. This study revealed that Campinas has high coverage of cervical and breast cancer screening, meeting national targets, but low prevalence of early detection tests for colorectal cancer. The results showed small social inequalities in relation to Pap smears and mammograms, but very high social inequalities in colorectal cancer screenings.

These results highlight the need for public policies that promote advances in social and health equity and in the development of organized cancer screening programs, to ensure access to early detection tests for all social segments of the population, promoting the reduction of morbidity and mortality associated with cancer.
