# Supplementary material for: Prevalence and social inequalities in the use of cancer screening tests in Campinas, Brazil (ISACamp 2014/15)
Source: Rev Bras Epidemiol. 2025 Aug 4;28:e250043. doi: 10.1590/1980-549720250043 (PMC12321152; doi:10.1590/1980-549720250043)
Supplement: Translation

# Translation: Prevalência e desigualdades sociais da realização de exames de detecção precoce de neoplasias no município de Campinas, SP (ISACamp 2014/15)

**DOI:** 10.1590/1980-549720250043.2

**Authors:** Bianca Gastaldon Lima, Marilisa Berti de Azevedo Barros

**Affiliations:** I Universidade Estadual de Campinas, Faculdade de Ciências Médicas, Departamento de Saúde Coletiva - Campinas (SP), Brasil.

**Keywords:** Programas de rastreamento, Neoplasias, Saúde do homem, Saúde da mulher

## Abstract

**Objetivo:**

Analisar a prevalência da realização de exames de detecção precoce de câncer do colo uterino, mama, próstata e colorretal na população de Campinas (SP) e a presença de desigualdades sociais no acesso.

**Métodos:**

Estudo transversal de base populacional que utilizou dados do Inquérito de Saúde do Município de Campinas 2014/15. As variáveis dependentes foram a realização de Papanicolaou, mamografia, antígeno prostático específico (PSA), sangue oculto nas fezes (SOF) e colonoscopia/sigmoidoscopia, nas faixas etárias recomendadas. As variáveis independentes incluíram sexo, idade, escolaridade, renda, raça/cor da pele e plano de saúde. Estimaram-se prevalências e razões de prevalência ajustadas por sexo e idade, por meio de regressão de Poisson. As análises foram realizadas no Stata 14, considerando-se os pesos amostrais.

**Resultados:**

As prevalências de mamografia (77,7%) e Papanicolaou (87,8%) atingiram as metas do Ministério da Saúde, enquanto as de SOF (22,3%) e colonoscopia/sigmoidoscopia (21,5%) foram baixas, e a de PSA nos últimos 3 anos foi de 55,2%. A prevalência de realização dos exames foi maior nos segmentos com maiores escolaridade e renda e com plano de saúde, e as desigualdades variaram conforme o exame. Pessoas com plano de saúde, por exemplo, apresentaram prevalências de Papanicolaou e colonoscopia/sigmoidoscopia, 11 e 162% maiores que as sem plano. Desigualdade por raça/cor foi observada somente na mamografia.

**Conclusão:**

Os resultados evidenciaram cobertura elevada e menores desigualdades para Papanicolaou e mamografia e baixa realização com importantes desigualdades para SOF e colonoscopia/sigmoidoscopia. Os achados indicam a importância do monitoramento da cobertura desses exames e a necessidade de políticas públicas que ampliem a equidade no acesso.

## INTRODUÇÃO

Nas últimas décadas, as neoplasias se destacaram-se como a segunda maior causa de morte global, atrás apenas das doenças cardiovasculares^1^ ^,^ ^2^. Em alguns países da Europa e no Canadá, já são a principal causa de mortalidade^3^.

Por causa da alta letalidade, a detecção precoce é essencial e pode ser feita por rastreamento, que aplica testes em indivíduos assintomáticos para identificar o câncer em estágio inicial, permitindo tratamentos menos invasivos e maior sobrevida^4^, no entanto o rastreamento envolve riscos, como falsos positivos, falsos negativos, excesso de tratamento e sobrediagnóstico^5^.

A Organização Mundial da Saúde (OMS) e a International Agency for Research on Cancer (IARC) recomendam o rastreamento para câncer do colo do útero, mama e colorretal, em razão das fortes evidências de benefícios^6^ ^,^ ^7^. Já para o câncer de próstata, não há recomendação dessas instituições para rastreamento de rotina em homens assintomáticos, dada a insuficiência de evidências sobre os riscos e benefícios do teste de antígeno prostático específico (PSA)^5^ ^,^ ^6^ ^,^ ^7^ ^,^ ^8^.

Estudos mostram que a prevalência dos exames de rastreamento varia entre países e há desigualdades sociais no acesso^9^ ^,^ ^10^ ^,^ ^11^ ^,^ ^12^ ^,^ ^13^ ^,^ ^14^ ^,^ ^15^ ^,^ ^16^ ^,^ ^17^ ^,^ ^18^ ^,^ ^19^ ^,^ ^20^ ^,^ ^21^. No Brasil, a realização dos exames de colo do útero e mama tem aumentado, mas com desigualdade entre grupos socioeconômicos^20^ ^,^ ^21^ ^,^ ^22^ ^,^ ^23^ ^,^ ^24^ ^,^ ^25^ ^,^ ^26^.

Diante da importância da detecção precoce e das disparidades no acesso, este estudo analisou a prevalência dos exames Papanicolaou, mamografia, PSA, sangue oculto nas fezes (SOF) e sigmoidoscopia/colonoscopia na população de Campinas (SP) e as desigualdades sociais presentes no acesso a esses exames.

## MÉTODOS

Este é um estudo transversal de base populacional desenvolvido com dados do Inquérito de Saúde do Município de Campinas (ISACamp) 2014/2015, que visa monitorar indicadores de saúde e uso de serviços em três grupos etários: adolescentes, adultos e idosos. O inquérito foi realizado em uma amostra da população não institucionalizada residente em domicílios particulares, na área urbana do município de Campinas.

A amostragem foi probabilística, estratificada, por conglomerados e realizada em dois estágios. No primeiro estágio foram sorteados 70 setores censitários, no segundo, domicílios nesses 70 setores para compor amostras independentes de 1.000 adolescentes (10-19 anos), 1.400 adultos (20-59 anos) e 1.000 idosos (60 anos ou mais).

Esses tamanhos de amostra permitem estimar uma proporção de 0,5, com nível de significância de 95%, erro amostral de 4 ou 5 pontos percentuais, considerando um efeito do delineamento de 2. O número de domicílios sorteados para cada domínio de idade foram respectivamente 3.119, 1.029 e 3.161. A coleta das informações do ISACamp 2014/15 foi realizada, com o auxílio de dispositivos *tablet*, por entrevistadores treinados e supervisionados. O questionário utilizado é composto de 12 blocos temáticos, com a maior parte das questões fechadas, e aplicado diretamente ao indivíduo selecionado.

No presente estudo foram utilizados apenas dados de adultos e idosos, nas faixas etárias recomendadas para os exames de detecção precoce analisados.

As variáveis dependentes utilizadas neste estudo foram:

- Realização do exame de Papanicolaou para rastreamento do câncer do colo do útero. Analisou-se sua realização nos três anos anteriores à entrevista em mulheres de 25 a 64 anos, faixa etária preconizada pelo Ministério da Saúde;
- Realização da mamografia para rastreamento do câncer de mama. Analisou-se sua realização nos dois anos anteriores à entrevista em mulheres de 40 a 69 anos, faixa etária preconizada pela Secretaria Municipal de Campinas, e de 50 a 69 anos, preconizada pelo Ministério da Saúde;
- Realização do exame de PSA para detecção do câncer de próstata. Analisaram-se duas variáveis: realização do exame nos três anos anteriores à entrevista e ao menos uma vez na vida, em homens com 50 anos ou mais, conforme recomendação da Sociedade Brasileira de Urologia;
- Realização do exame de SOF para rastreamento do câncer colorretal nos dois anos anteriores à entrevista, em homens e mulheres com 50 anos ou mais, conforme recomendação da Sociedade Brasileira de Coloproctologia e do Ministério da Saúde;
- Realização de sigmoidoscopia ou colonoscopia ao menos uma vez na vida para rastreamento do câncer colorretal, em homens e mulheres com 50 anos ou mais, conforme recomendação da Sociedade Brasileira de Coloproctologia e do Ministério da Saúde.

As variáveis independentes analisadas foram: sexo (masculino e feminino), faixa etária (em anos), escolaridade (em anos de estudo concluídos: 0 a quatro, cinco a 11 e 12 anos ou mais), filiação a plano privado de saúde (tem ou não tem), raça/cor de pele autorreferida (brancos, pretos e pardos - amarelos e indígenas não entraram na análise por causa do pequeno número na amostra) e renda mensal familiar *per capita* (em salários mínimos: <1, 1 a <2 e 2 ou mais).

Os dados foram analisados no Stata 14 (StataCorp, College Station, TX, Estados Unidos da América) usando o módulo *survey*, adequado para amostras complexas. Estimaram-se as prevalências das práticas preventivas com intervalo de confiança de 95% (IC95%). As associações entre variáveis foram verificadas pelo teste χ^2^ de Pearson (*p*≤0,05). Razões de prevalência entre segmentos sociodemográficos foram estimadas e comparadas por regressão múltipla de Poisson com variação robusta, ajustada por sexo e idade.

O ISACamp 2014/15 foi aprovado pelo Comitê de Ética da Faculdade de Ciências Médicas da Universidade Estadual de Campinas (parecer nº 409.714/2013), assim como o presente projeto (parecer nº 6.983.946/2024).

### Declaração de disponibilidade de dados

Os dados utilizados neste estudo estão disponíveis mediante solicitação ao autor correspondente, considerando os critérios de privacidade e confidencialidade dos participantes, em conformidade com as normas éticas aplicáveis.

## RESULTADOS

A Tabela 1 apresenta as prevalências de realização dos exames de detecção precoce e o número de pessoas da amostra (n) na faixa etária recomendada. Os resultados mostram que em Campinas a prevalência do Papanicolaou (nos três anos prévios à entrevista) foi de 87,8% entre as mulheres de 25 a 64 anos; e a mamografia (nos dois anos prévios), de 80,5% nas mulheres de 40 a 69 anos e de 77,7% nas de 50 a 69 anos.

**Tabela 1.** Prevalências (IC95%) de realização de exames para detecção precoce de neoplasias. Inquérito de Saúde do Município de Campinas 2014/15.

Exames realizados	Faixa etária (anos)	n	Prevalência em % (IC95%)
Papanicolaou (nos últimos três anos)	25 a 64	639	87,8 (84,2-90,7)
Mamografia (nos últimos dois anos)	40 a 69	548	80,5 (75,1-84,9)
Mamografia (nos últimos dois anos)	50 a 69	315	77,7 (71,5-82,9)
PSA (nos últimos três anos)	50 ou mais	499	55,2 (49,7-60,5)
PSA (um exame na vida)	50 ou mais	499	80,1 (75,0-84,4)
SOF (nos últimos dois anos)	50 ou mais		
Ambos os sexos		1.228	22,3 (17,8-27,5)
Homens		499	22,2 (17,1-28,3)
Mulheres		729	22,4 (17,5-28,2)
Sigmoidoscopia/colonoscopia (um exame na vida)	50 ou mais		
Ambos os sexos		*1.217	21,5 (17,9-25,5)
Homens		495	17,6 (13,0-23,3)
Mulheres		722	24,6 (20,5-29,2

IC95%: intervalo de confiança de 95%; PSA: antígeno prostático específico; SOF: sangue oculto nas fezes; n: número total de indivíduos em cada faixa etária, independentemente da realização dos exames; *11 pessoas não responderam.

Em Campinas, 55,2% dos homens com 50 anos ou mais realizaram o exame de PSA nos três anos anteriores à entrevista, e 80,1% ao menos uma vez na vida. Entre a população de 50 anos ou mais, 22,3% realizaram o exame de SOF nos dois anos prévios, sem diferença das prevalências entre os sexos, e 21,5% fizeram colonoscopia/sigmoidoscopia ao menos uma vez na vida, com prevalência significativamente maior no sexo feminino (24,6%) em relação ao masculino (17,6%) (Tabela 1), com razão de prevalência (RP) ajustada por idade de 1,38 (1,02-1,85).

Nas análises das diferenças de prevalência segundo características sociodemográficas, observou-se maior prevalência de realização dos exames de rastreamento entre pessoas com plano de saúde. A desigualdade foi pequena para o Papanicolaou (RP=1,11) e mamografia (RP=1,20-1,29), intermediária para PSA (RP=1,47) e SOF (RP=1,67) e muito elevada para colonoscopia/sigmoidoscopia (RP=2,62) (Tabela 2).

**Tabela 2.** Prevalências e razões de prevalências* (IC95%) da realização de exames para detecção precoce segundo ter plano privado de saúde. Inquérito de Saúde do Município de Campinas 2014/15.

Exames realizados	Não tem plano	Tem plano	RP (IC95%)**
Papanicolaou (nos últimos três anos)	83,0 (77,1-87,5)	92,9 (88,5-95,6)	1,11 (1,04-1,20)
Mamografia^a^ (nos últimos dois anos)	73,3 (66,6-79,1)	87,9 (80,9-92,6)	1,20 (1,08-1,32)
Mamografia^b^ (nos últimos dois anos)	68,0 (60,0-75,0)	88,0 (79,8-93,1)	1,29 (1,13-1,47)
PSA (nos últimos três anos)	46,0 (39,0-53,0)	68,0 (58,4-76,2)	1,47 (1,20-1,78)
PSA (um exame na vida)	73,7 (66,9-79,6)	89,2 (82,5-93,6)	1,20 (1,09-1,32)
SOF (nos últimos dois anos)			
Ambos os sexos	16,9 (12,3-22,9)	28,5 (21,7-36,4)	1,67 (1,13-2,46)
Homens	18,4 (12,7-25,8)	27,4 (20,1-36,2)	1,46 (0,96-2,22)
Mulheres	15,5 (10,8-21,7)	29,2 (21,2-38,7)	1,87 (1,16-3,01)
Sigmoidoscopia ou colonoscopia (um exame na vida)			
Ambos os sexos	12,2 (9,5-15,6)	32,3 (25,8-39,5)	2,62 (1,88-3,65)
Homens	10,8 (7,5-15,3)	27,1 (18,6-37,7)	2,45 (1,54-3,90)
Mulheres	13,4 (10,0-17,8)	35,6 (28,2-43,8)	2,64 (1,78-3,92)

*Ajustadas por sexo e idade; ^a^mulheres de 40 a 69 anos; ^b^mulheres de 50 a 69 anos; IC95%: intervalo de confiança de 95%; PSA: antígeno prostático específico; SOF: sangue oculto nas fezes; RP: razão de prevalência; **população que tem plano / população sem plano e população branca / população preta e parda.

A Tabela 3 mostra que as prevalências são significativamente mais altas entre indivíduos com 12 ou mais anos de escolaridade, sem diferença estatisticamente significativa entre os de escolaridade intermediária e os de menor escolaridade, exceto para o PSA (um exame na vida), que foi 16% mais elevado entre aqueles com cinco a 11 anos de estudo em comparação aos com menos de quatro anos. As maiores desigualdades ocorreram nos exames de SOF (RP=2,20) e colonoscopia/sigmoidoscopia (RP=2,86).

**Tabela 3.** Prevalências e razões de prevalências* (IC95%) da realização de exames para detecção precoce segundo escolaridade em anos. Inquérito de Saúde do Município de Campinas 2014/15.

Exames realizados	0 a quatro anos de escolaridade	Cinco a 11 anos de escolaridade	12 anos ou mais de escolaridade	RP (IC95%) (5 a 11/<4)	RP (IC95%) (12 ou +/<4)
Papanicolaou (nos últimos três anos)	81,7 (73,1-88,0)	86,9 (82,3-90,5)	92,8 (86,5-96,3)	1,10 (0,99-1,22)	1,18 (1,07-1,31)
Mamografia^a^ (nos últimos dois anos)	71,4 (62,7-78,8)	80,6 (73,4-86,2)	92,4 (81,3-97,1)	1,12 (0,97-1,29)	1,28 (1,08-1,50)
Mamografia^b^ (nos últimos dois anos)	68,3 (59,8-75,8)	79,1 (70,4-85,7)	94,1 (82,3-98,2)	1,15 (0,97-1,34)	1,36 (1,15-1,60)
PSA (nos últimos três anos)	54,2 (47,0-61,2)	51,8 (43,8-59,6)	64,5 (49,2-77,4)	1,12 (0,90-1,38)	1,38 (1,06-1,79)
PSA (um exame na vida)	75,3 (67,2-82,0)	79,0 (71,2-85,1)	92,5 (79,7-97,5)	1,16 (1,01-1,34)	1,36 (1,16-1,58)
SOF (nos últimos dois anos)					
Ambos os sexos	20,4 (15,5-26,3)	18,0 (13,0-24,3)	37,6 (24,7-52,5)	1,05 (0,75-1,45)	2,20 (1,42-3,44)
Homens	21,4 (15,9-29,1)	17,5 (11,5-25,6)	33,7 (20,7-49,8)	1,12 (0,69-1,83)	2,15 (1,25-3,67)
Mulheres	19,6 (14,5-26,0)	18,4 (12,7-25,9)	41,5 (22,3-63,6)	1,01 (0,64-1,59)	2,31 (1,30-4,10)
Sigmoidoscopia ou colonoscopia (um exame na vida)					
Ambos os sexos	19,1 (14,8-24,1)	14,1 (11,1-17,7)	46,1 (32,2-60,7)	0,87 (0,63-1,20)	2,86 (1,88-4,36)
Homens	15,5 (11,5-20,6)	9,6 (5,6-15,8)	39,7 (23,8-58,0)	0,9 (0,51-1,55)	3,61 (2,15-6,06)
Mulheres	21,4 (15,8-28,2)	18,0 (13,2-23,9)	52,8 (34,1-70,7)	0,89 (0,57-1,38)	2,63 (1,61-4,31)

*Ajustadas por sexo e idade; ^a^mulheres de 40 a 69 anos; ^b^mulheres de 50 a 69 anos; IC95%: intervalo de confiança de 95%; PSA: antígeno prostático específico; SOF: sangue oculto nas fezes; RP: razão de prevalência.

Na Tabela 4, são apresentadas as RP dos exames segundo raça/cor. Observa-se que não há diferenças significativas para quatro dos exames analisados, e apenas a prevalência de mamografia foi significativamente maior entre mulheres brancas de 40 a 69 anos (RP=1,17) em comparação à população negra.

**Tabela 4.** Prevalências e razões de prevalências* (IC95%) da realização de exames para detecção precoce segundo raça/cor. Inquérito de Saúde do Município de Campinas 2014/15.

Exames realizados	Pretos e pardos	Brancos	RP (IC95%)**
Papanicolaou (nos últimos três anos)	85,3 (80,1-89,4)	89,2 (84,9-92,5)	1,04 (0,98-1,11)
Mamografia^a^ (nos últimos dois anos)	72,4 (62,0-80,8)	84,1 (78,6-88,4)	1,17 (1,02-1,33)
Mamografia^b^ (nos últimos dois anos)	71,1 (60,5-79,9)	81,0 (74,0-86,5)	1,14 (0,98-1,32)
PSA (nos últimos três anos)	53,2 (43,4-62,7)	56,4 (49,8-62,9)	1,07 (0,86-1,35)
PSA (um exame na vida)	77,6 (65,7-86,2)	81,7 (75,3-86,8)	1,05 (0,90-1,24)
SOF (nos últimos dois anos)			
Ambos os sexos	20,6 (15,4-26,8)	23,2 (17,9-29,4)	1,12 (0,82-1,53)
Homens	19,8 (13,4-28,4)	23,1 (17,3-30,1)	1,19 (0,81-1,75)
Mulheres	21,1 (14,5-29,6)	23,2 (17,0-30,7)	1,09 (0,68-1,75)
Sigmoidoscopia ou colonoscopia (um exame na vida)			
Ambos os sexos	20,1 (15,1-26,3)	22,0 (17,6-27,1)	1,08 (0,76-1,55)
Homens	18,5 (10,9-29,8)	17,2 (11,8-24,4)	0,95 (0,49-1,82)
Mulheres	21,3 (15,5-28,6)	25,8 (20,5-31,9)	1,20 (0,80-1,81)

*Ajustadas por sexo e idade; ^a^mulheres de 40 a 69 anos; ^b^mulheres de 50 a 69 anos; IC95%: intervalo de confiança de 95%; PSA: antígeno prostático específico; SOF: sangue oculto nas fezes; RP: razão de prevalência; **população que tem plano / população sem plano e população branca / população preta e parda.

A Tabela 5 mostra maior prevalência de todos os exames de detecção precoce entre indivíduos com renda *per capita* familiar superior a dois salários mínimos, destacando-se os exames de colonoscopia/sigmoidoscopia, 2,5 vezes mais prevalentes. Indivíduos com renda de um a dois salários mínimos apresentaram maior prevalência de mamografia (mulheres de 40 a 69 anos) e colonoscopia/sigmoidoscopia em comparação aos com renda inferior a um salário mínimo.

**Tabela 5.** Prevalências e razões de prevalências* (IC95%) da realização de exames para detecção precoce segundo renda familiar *per capita* em salários mínimos. Inquérito de Saúde do Município de Campinas 2014/15.

Exames realizados	<1 SM	1 a <2 SM	2 ou mais	RP (IC95%) (1 a <2 SM /<1)	RP (IC95%) (2 ou +/<1)
Papanicolaou (nos últimos três anos)	82,7 (75,7-88,0)	89,3 (83,4-93,2)	92,2 (87,9-95,1)	1,08 (0,98-1,18)	1,11 (1,03-1,2)
Mamografia^a^ (nos últimos dois anos)	73,4 (66,2-79,6)	80,5 (73,0-86,3)	86,5 (77,6-92,2)	1,10 (1,0-1,21)	1,19 (1,06-1,34)
Mamografia^b^ (nos últimos dois anos)	66,2 (56,7-74,6)	75,5 (66,2-82,9)	88,3 (78,7-93,9)	1,14 (0,97-1,34)	1,34 (1,16-1,55)
PSA (nos últimos três anos)	46,7 (36,8-56,9)	54,0 (44,1-63,6)	63,2 (53,5-71,9)	1,15 (0,86-1,52)	1,36 (1,04-1,77)
PSA (um exame na vida)	72,4 (60,8-81,7)	78,8 (68,8-86,2)	87,2 (79,2-92,4)	1,08 (0,89-1,31)	1,20 (1,01-1,42)
SOF (nos últimos dois anos)					
Ambos os sexos	16,3 (11,8-22,2)	21,0 (15,3-28,0)	28,0 (20,1-37,5)	1,29 (0,9-1,8)	1,73 (1,11-2,7)
Homens	14,4 (8,2-23,8)	23,5 (15,7-33,6)	25,7 (18,7-34,1)	1,60 (0,91-2,82)	1,81 (1,03-3,17)
Mulheres	17,7 (11,9-25,4)	18,7 (13,0-26,2)	29,8 (19,8-42,3)	1,06 (0,64-1,73)	1,69 (0,92-3,07)
Sigmoidoscopia ou colonoscopia (um exame na vida)					
Ambos os sexos	11,6 (8,0-16,4)	21,4 (15,8-28,4)	28,8 (21,7-37,2)	1,85 (1,14-3,0)	2,50 (1,58-3,98)
Homens	6,8 (3,31-13,3)	16,8 (10,5-25,7)	25,2 (17,6-34,7)	2,41 (1,08-5,36)	3,78 (1,79-8,00)
Mulheres	14,9 (9,8-21,9)	25,4 (18,8-33,3)	31,7 (22,1-43,1)	1,70 (1,00-2,90)	2,13 (1,19-3,78)

*Ajustadas por sexo e idade; ^a^mulheres de 40 a 69 anos; ^b^mulheres de 50 a 69 anos; IC95%: intervalo de confiança de 95%; PSA: antígeno prostático específico; SOF: sangue oculto nas fezes; RP: razão de prevalência; SM: salário mínimo.

## DISCUSSÃO

Os resultados deste estudo indicaram que as prevalências dos exames de Papanicolaou e mamografia em Campinas atingiram as metas recomendadas pelo Ministério da Saúde. As prevalências de SOF e colonoscopia/sigmoidoscopia foram inferiores às de outras prevalências. As análises revelaram desigualdades sociais no acesso aos exames, com maior prevalência nos indivíduos com níveis mais elevados de escolaridade e renda e com plano de saúde. Foram observadas disparidade de raça-cor na realização de mamografia, com maior prevalência entre as mulheres brancas, e disparidade entre os sexos na realização de colonoscopia/sigmoidoscopia, com maior prevalência entre as mulheres em comparação aos homens (RP=1,38).

A cobertura de rastreamento para o câncer do colo do útero em Campinas foi de 87,8%, atingindo a meta de 85% preconizada pelo Ministério da Saúde^27^. No município de São Paulo (SP), no mesmo período, a cobertura foi similar, alcançando 89,6%^23^. Dados da Pesquisa Nacional de Saúde (PNS) mostraram aumento da taxa de realização do Papanicolaou das mulheres brasileiras de 78,7% em 2013 para 81,3% em 2019^20^, mas com percentual ainda um pouco abaixo do preconizado pelo Ministério da Saúde.

Dados de estudos realizados em alguns países desenvolvidos mostram prevalências de realização desse exame similares ou inferiores às da população brasileira. No Reino Unido, a prevalência de realização do exame de Papanicolaou também entre mulheres de 25 a 64 anos foi de 71% em 2018^10^, e nos Estados Unidos, entre mulheres de 21 a 65 anos, foi de 80% em 2018^9^.

Estudos prévios realizados no município de Campinas permitem observar a persistência da realização do exame de Papanicolaou em patamar elevado e com tendência a discreto aumento: 83,3% em 2001/2002, 86,2% em 2008/09 (ambos em mulheres de 20 a 60 anos), e 87,8% no presente estudo (em mulheres de 25 a 64 anos)^21^ ^,^ ^22^.

No presente estudo, as prevalências de realização do exame foram 11% maiores no segmento de maior renda, 18% no de maior escolaridade e 11% nas mulheres com plano de saúde, em comparação às categorias de referência. As desigualdades observadas na população brasileira, com os dados da PNS de 2019, foram: 24% maior no segmento com mais anos de estudo, 29% maior no melhor nível de renda e 20% maior nas mulheres que possuem plano de saúde^20^, um pouco superiores, portanto, às observadas em Campinas.

Estudo realizado em 22 países europeus verificou que a prevalência do Papanicolau foi na média 28% superior nos segmentos das mulheres com maior escolaridade, variando entre 7,7% em Luxemburgo e 44% na Croácia^12^. As desigualdades segundo escolaridade observadas em Campinas e no Brasil se situam, assim, abaixo da média detectada para esses países europeus.

Em Campinas, estudo desenvolvido com dados do inquérito de 2008/2009 não havia detectado desigualdades sociais no acesso ao Papanicolaou em mulheres na faixa de 20 a 60 anos^22^. O ressurgimento da desigualdade mostra a necessidade do monitoramento não só da cobertura dos exames, como também da presença e do tamanho das desigualdades sociais na realização deles.

Para o exame de mamografia, o Ministério da Saúde recomenda cobertura de 70% para mulheres na faixa etária de 50 a 69 anos^27^. No município de Campinas, o presente estudo detectou prevalência de 77,7% nessa faixa etária, superando a meta recomendada, o que também foi observado no município de São Paulo, que detectou em 2015 prevalência de 73,8%^23^. Dados da PNS de 2013 mostram que 79,4% das mulheres de 40 a 70 anos haviam feito mamografia nos dois anos prévios à entrevista, porém, na faixa etária recomendada pelo Ministério da Saúde, a prevalência foi de 58%^25^.

Estudo realizado em 2010 em 22 países europeus revelou que a prevalência de realização da mamografia nos países com rastreamento organizado variou de 69,8% no Reino Unido a 87,9% na Finlândia e, entre os quatro países sem rastreamento organizado, de 38,1% na Letônia a 50,5% na República Checa^12^. Nos Estados Unidos a prevalência de mamografias foi de 78% em 2020, variando de 66 a 87% entre os estados americanos^11^.

Comparando-se a prevalência de mamografia de Campinas com os dados de outros países, constatou-se que, mesmo sem o rastreio organizado para o câncer de mama, as prevalências são similares e até superiores a alguns países que contam com o rastreamento organizado.

No presente estudo, as prevalências de realização do exame na faixa de 50 a 69 anos foram: 36% maiores no segmento de maior escolaridade, 34% no de maior renda e 29% entre as mulheres com plano de saúde em comparação às categorias de referência. Dados da PNS de 2013 mostram prevalência duas vezes maior no segmento de maior escolaridade e 11% superior nas mulheres com plano de saúde^25^ ^,^ ^28^. As desigualdades na realização de mamografias, medidas pela escolaridade, variam significativamente entre alguns países europeus: 46,4% na Grécia, 47,9% na Croácia e 82,8% na República Tcheca^12^, que apresentam desigualdades superiores à observada em Campinas.

O rastreamento de rotina do câncer de próstata por meio do exame de PSA não é recomendado para homens assintomáticos pela OMS e pela IARC em razão da falta de evidências claras sobre os benefícios e riscos^6^ ^,^ ^7^. O Ministério da Saúde também desaconselha o rastreamento, enquanto a Sociedade Brasileira de Urologia recomenda que homens com os 50 anos ou mais discutam com seus urologistas a possibilidade de realizar exames de detecção precoce do câncer de próstata^5^ ^,^ ^29^.

O presente estudo detectou a prevalência de realização do exame PSA nos três anos anteriores à pesquisa de 55,2%, em homens com 50 anos ou mais, e de 80,1% para a realização do exame ao menos uma vez na vida. Estudo conduzido no município de São Paulo, no mesmo período, verificou que 63,2% dos homens com 40 anos ou mais havia realizado o PSA ao menos uma vez na vida^23^. A prevalência de realização do exame de PSA ao menos uma vez na vida foi de 70% entre homens de 55 a 69 anos, em estudo realizado na Holanda^16^, e de 44,2%, na faixa de 40 anos ou mais, em pesquisa realizada em Portugal^17^. Embora sejam analisadas faixas etárias diferentes, a realização de PSA uma vez na vida em Campinas se mostra superior à desses países e à observada no município de São Paulo.

No presente estudo, a realização do PSA nos três anos anteriores à entrevista foi 36% maior entre os homens de maior renda, 38% entre os de maior escolaridade e 47% entre aqueles com plano de saúde. Estudo realizado no município de São Paulo constatou que, em 2015, a realização do PSA uma vez na vida, em homens de 40 anos ou mais, foi 48% mais elevada no segmento de maior escolaridade^23^, desigualdade que não difere significativamente da que foi observada em Campinas.

Os resultados deste estudo indicam que, mesmo sem diretrizes governamentais para o rastreamento populacional do câncer de próstata, uma parcela significativa dos homens em Campinas realizou o exame de PSA, superando as prevalências observadas em países europeus e no município de São Paulo, no entanto persistem desigualdades no acesso ao exame, com taxas mais elevadas entre os homens de maior nível socioeconômico.

O Ministério da Saúde recomenda a realização dos exames de rastreamento para o câncer colorretal, mas não tem um programa de rastreamento organizado nem estabelece metas específicas de cobertura para cada exame^5^ ^,^ ^27^. Diretrizes da União Europeia estabelecem cobertura acima de 65% para exame de SOF a cada dois anos como garantia de qualidade na triagem de câncer colorretal^15^.

Os resultados deste estudo mostraram prevalências de 22,3% para o teste de SOF realizado nos dois anos anteriores à entrevista e de 21,5% para colonoscopia/sigmoidoscopia (um exame na vida) entre indivíduos de 50 anos ou mais. Dados de 2007 de países europeus revelaram que as taxas de realização do exame de SOF, utilizando teste imunoquímico fecal a cada dois anos, variavam de 42% na França a 70,8% na Finlândia^14^. Um estudo de 2019 desenvolvido com dados da National Health Interview Survey revelou que, entre os idosos de 65 a 75 anos nos Estados Unidos, 19,2% realizaram colonoscopia e 6% fizeram o teste de SOF no ano anterior à pesquisa^13^.

Uma revisão sistemática que analisou 96 artigos de 14 países encontrou ampla variação na participação da população nos exames de rastreamento do câncer colorretal, com prevalências de sigmoidoscopia entre 7 e 76,1%, de SOF entre 2,3 e 68,7% e de colonoscopia após um resultado positivo no exame de SOF entre 72,9 e 92,6%^19^.

A comparação das prevalências entre os estudos é dificultada pelas diferenças nas faixas etárias e no período de realização dos exames e também pela ausência de estudos nacionais com análises de prevalências de realização dos exames de detecção precoce do câncer colorretal, mas a comparação com dados de outros países sinaliza a baixa cobertura desses exames no município de Campinas.

Outro dado relevante foi a presença de elevadas desigualdades no acesso a esses exames. A realização do teste de SOF foi 73% mais frequente entre indivíduos de maior renda, e a de colonoscopia/sigmoidoscopia, 150% mais comum nesse grupo. Em termos de escolaridade, o acesso ao SOF foi 120% maior entre os mais escolarizados, enquanto a colonoscopia/sigmoidoscopia apresentou diferença de 186%. Nos indivíduos com plano de saúde, a prevalência de realização do SOF foi 67% maior, e a de colonoscopia/sigmoidoscopia, 162% superior.

A revisão sistemática que teve como foco as desigualdades sociais na participação de programas de rastreamento do câncer colorretal verificou que, em geral, os segmentos da população com maior nível de escolaridade e renda apresentam maior participação nos exames de SOF e colonoscopia^19^. Estudo dinamarquês de 2010 revelou razão de *odds* de 1,38 na realização de SOF entre aqueles com ensino superior em comparação com os de menor escolaridade^30^. Apesar da diferença no período analisado e na forma de medir a associação, a desigualdade observada na Dinamarca foi bem inferior à observada em Campinas no presente estudo.

Entre os resultados deste estudo, foi constatado que a realização de colonoscopia/sigmoidoscopia foi 38% maior nas mulheres em relação aos homens e sem diferença entre os sexos na realização do SOF. Diferentemente do nosso estudo, artigos de outros países mostraram prevalência superior na realização de SOF nas mulheres em comparação aos homens^19^ ^,^ ^30^. Na metanálise já mencionada, a maioria dos estudos detectou maior participação das mulheres, em comparação aos homens, nos exames de rastreamento do câncer colorretal, o que é atribuído ao maior uso que elas fazem dos serviços de saúde e maior familiaridade com programas de rastreamento^19^. Enquanto nas mulheres o autocuidado está mais presente, para prevenir sofrimento pessoal e familiar, nos homens é pobre a percepção de vulnerabilidade^19^.

Os resultados do presente estudo revelaram baixa cobertura dos exames de detecção precoce do câncer colorretal em Campinas e significativas desigualdades socioeconômicas na realização deles, o que evidencia a necessidade de estruturação adequada dos serviços de saúde para a oferta ampliada dos exames e redução das desigualdades de acesso.

O presente estudo é relevante por analisar, de forma conjunta, diversos exames de detecção precoce de neoplasias, permitindo comparações quanto às coberturas populacionais atingidas e à magnitude das desigualdades sociais associadas. Entre as limitações do estudo, deve-se destacar a possibilidade de viés de memória e informação, uma vez que os dados foram obtidos por entrevistas, e os entrevistados podem se confundir quanto ao tempo em que realizaram o exame e ter respondido que realizaram o exame por considerarem esta a melhor resposta, conforme apontado em outros estudos^24^ ^,^ ^25^ ^,^ ^26^.

Para mitigar esses vieses, o presente estudo foi conduzido com rigor metodológico, incluindo a aplicação de questionários padronizados e treinamento específico dos entrevistadores para assegurar a clareza das perguntas e reduzir interpretações ambíguas. Sendo este um estudo transversal, também é preciso considerar que as perguntas sobre a realização dos exames se referem aos dois ou três anos anteriores à entrevista ou a alguma vez na vida, a questão sobre a renda diz respeito ao mês anterior e aquela sobre escolaridade e ter plano de saúde ao momento da entrevista.

Este estudo aponta para a importância do monitoramento da realização dos exames de detecção precoce das neoplasias para verificar em que grau estão sendo atingidas as metas estabelecidas. Ao evidenciar e mensurar as disparidades no acesso ao rastreamento entre segmentos socioeconômicos, possibilita identificar os exames com menor ou maior grau de desigualdade de acesso e os segmentos da população em maior risco de diagnóstico tardio e orientar na direção de ações que promovam avanços na equidade em saúde. O estudo também gera informações para futuras comparações com dados pós-pandemia.

Este estudo revelou que Campinas apresenta alta cobertura de rastreamento para câncer do colo uterino e de mama, atingindo as metas nacionais, mas baixa prevalência de exames de detecção precoce de câncer colorretal. Os resultados mostraram desigualdades sociais de pequena magnitude em relação ao Papanicolau e à mamografia, porém desigualdades sociais muito elevadas nos exames para o câncer colorretal.

Tais resultados evidenciam a necessidade de políticas públicas que promovam avanços na equidade social e em saúde e no desenvolvimento de programas organizados de rastreamento de neoplasias, para assegurar o acesso aos exames de detecção precoce a todos os segmentos sociais da população, promovendo a redução da morbimortalidade associada ao câncer.
